# Case of an Enlarged and Tuberculated Liver, a Part of Which Having Formed a Pulsating Tumour in the Epigastrium, Was Mistaken for an Aneurism; Accompanied with an Exquisitely Painful Enlargement of the Hip, the True Nature of Which, Also, Could Not Be Ascertained during Life; but Was Afterwards Found to Have Been Occasioned by an Extensive Caries of the Joint

**Published:** 1807-12

**Authors:** James Millman Coley

**Affiliations:** Member of the Royal College of Surgeons in London, and Surgeon in Bridgnorth


### Medical and Phyfical Journal.

VOL. XVIII.] DECEMBER, 1807. [NO. 106.

*Printed for R. PHILLIPS, by W. Thorne, Red Lion Court, Fleet Street, London*

### *Case of an enlarged and tuberculated Liver, a Part of which having formed a pulsating Tumour in the Epigastrium, was mistaken for an Aneurism; accompanied with an exquisitely painful Enlargement of the Hip, the true Nature of which, also, could not be ascertained during Life; but was afterwards found to have been occasioned by an extensive Caries of the Joint.* Communicated by JAMES MILLMAN COLEY, Member of the Royal College of Surgeons in London, and Surgeon in Bridgnorth.

SEPT. 2, 1806.—W. R. aged about 50, of a spare habit, and, till within a few years, accustomed to much vinous potation, complained of a pain at the stomach, to which he had been subject during the last six months. He remarked, at the same time, that about three years ago, he was severely crushed by a horse against a stall in a stable, which rendered him for some minutes unable to breathe. On recovering, he found the epigastrium bruised and painful; but he soon got perfectly well. Not conceiving this accident had any connection with the present complaint, which, from his description, appeared to be spasmodic, he was recommended to take pills of zinc and opium; to live temperately, and to observe some dietetic directions that were given him.

After this, he became affected with symptoms of *pyrosis*, for which were ordered suitable remedies.

The pain continued to increase, accompanied with soreness about the epigastrium; and on applying there a gentle pressure with the hand, a hard tumour was very distinctly felt. It was difficult to say what this tumour was; but, at this time, I imagined it to be a part of an enlarged and diseased liver.

Oct. 16.—On this and the preceding days, the patient noticed a pulsation in the tumour; which did not materially interest him, as he supposed it to have arisen from the heart having changed its situation, since he could not feel it beat against the side as usual. I felt the pulsating motion myself on this day, while he was standing in an erect posture, and found it to correspond with the artery at the wrist in point of frequency; but the action of the heart could not be perceived in its natural place of pulsation at the side. These circumstances led me to think that he had been mistaken in observing the motion of the heart on the left side formerly, and that it must have originally been misplaced; a circumstance that has not only been suspected during life, but has been ascertained on dissection afterwards. (Abernethy's Anatomical Lectures.) This idea, however, proved to be erroneous; since, on placing the body in a recumbent position, the vibration of the heart was readily discovered in the natural situation.

A fortnight after this time he remarked, that on walking hastily the pain was much increased; and that he was obliged to stop suddenly, being unable to proceed for several minutes. His respiration was not affected, and the pulse at the wrist was natural but weak.

At this period he was more minutely examined, in a consultation with my father and another medical friend; when we were all of opinion that the tumour itself was either aneurismal, or that aneurism existed in some artery within the cavity of the abdomen.

Dec. 18.—The tumour in the epigastrium had increased to the size of an adult fist; and the pulsation, which had now become strong, felt in every part of it, as though it were situated immediately under the integuments.

May, 1807.—Till this month, he continued in nearly the same situation; and was in the habit of using temperate exercise, to the extent of four miles in a day, once or twice in a week, without much inconvenience. He was now seized with a complaint, which confined him to his bed during the whole remainder of his life. It began with a spasmodic pain in the right hip, which was tender externally; and, in a few days, this tenderness extended down the thigh in the course of the *tensor vaginæ femoris*, accompanied with inflammation, swelling, and excruciating pain. At the same time, a fullness was perceivable within the capsule of the joint; and as the pain became more deep-seated, I suspected inflammation in this part, as well as in the fascia on the outside, on which account several leeches were applied at two or three different periods; but they did not contribute to lessen the pain or inflammation. Repeated blistering produced no better effect. The pain pain was at length so violent, as to require internal remedies. He took gr. jss. of opium, and gr. xv of cicuta, every eighth hour; and afterwards gr. vj. of opium at bedtime, which only disordered the head and stomach, affording him no relief. With as little success were afterwards administered large doses of camphor, with opium, guaiacum, and ammonia; also ol. terebinthinæ, and various other articles*. No applications relieved the pain through the whole course of his illness, but fomentations and poultices.

The hip, at length, became of an amazing size, and the thigh was observed to be much shorter than the other. The inflammation spreading downwards, was accompanied with œdema; and the whole leg and foot partook of the same kind of inflammatory tumefaction.

Three months having now elapsed from the period of his first being confined to bed, the same species of œdematous swelling, which existed in the right leg, had likewise formed in the left, reaching from the foot up to the pelvis. This swelling, particularly in some parts of it, was nearly as hard, shining, and tense, as the tumefaction which characterises *phlegmatia dolens;* and continued to increase, notwithstanding the exhibition of digitalis, mercury, and other very powerful diuretics.

During the period of his confinement till his death, the pulsation in the epigastrium grew daily more feeble, till at length it was scarcely to be felt; and, for the last two months, he made no complaint of pain or tenderness in that part. As he lay constantly on his back, I took frequent opportunities of examining the left side, and always found the heart beating in the natural situation, but as low down as between the sixth and seventh ribs. At last a mortification, purely from long-continued decumbiture, took place upon the back; and this ended in a large spreading ulceration, with which he died soon afterwards, on Sept. 25, 1807.

While the tumour in the epigastrium was the more important part of this man's complaints, we availed ourselves of opportunities of consulting the most intelligent of our medical friends. Among these may particularly be men-

* It was very extraordinary, that of the many medicines he took, the only one which was found capable of giving ease and producing sleep, was the inspissated juice of hyoscyamus, administered in large quantity; but this effect wore off in the course of three or four nights, although the dose was so much increased, as to induce a violent diarrhœa.

 tioned tioned Mr. Sandford, of Worcester, a surgeon of great scientific knowledge and experience, who took great pains to investigate the nature of the disease; and his opinion of the case was, that the pulsating swelling described, either constituted an aneurism, or that the pulsation was occasioned by a tumour or indurated gland, producing unequal pressure on some arterial branch; or that both affections might exist, and act upon each other*. The case was afterwards represented by my father to Dr. Darwin, of Shrewsbury, of whose very superior judgment he entertains the highest opinion; and the Doctor had afterwards an opportunity of examining the man personally. He was also of opinion, that the case was aneurism; and prescribed remedies for the management of him accordingly.

### Morbid Appearances discovered after the Death of the Patient.

### I. *Dissection of the Abdomen.*

The abdomen being laid open, the first thing of importance presenting itself was a large mass, partly white, partly brown, and of irregular surface; filling up the whole epigastrium, and extending preternaturally into the left hypochondrium. This was found to be part of the left lobe of the liver, the whole of which, together with the *lobulus Spigelii,* were so much diseased, as not to have the least resemblance to a liver in its healthy state. There were several morbid adhesions between this part and the diaphragm, which being separated, as well as the different ligamentous attachments naturally existing between them, exposed to view the *vena cava hepatica.* Over this vessel a ligature was applied, close to the tendinous centre of the diaphragm, for the purpose of preventing an effusion of blood; and this it did completely. I now got the liver fairly out of the abdomen, when it appeared two or three times as large as in a natural state. The whole of the left lobe was full of tubercles, the largest of which did not exceed in size a hen's egg; and some were as small as

* At the time W. R. was examined by Mr. S. it is somewhat remarkable, that he happened to have a patient labouring under what he conceived to be the same disease; and, by his request, the two patients were brought together, and were themselves entirely convinced of the supposed identity of their cases.

common common horse-beans. These, to the feel, were of various solidity, apparently in degree proportionate to the respective bulk of them; the smallest being incompressible with the fingers, while the largest were very soft. On the margin and concave surface of this lobe, and on the *lobulus Spigelii*, (which was also tuberculated) the minute vessels were gorged with blood of a dark crimson hue, and in some places approaching to black; indicating very active inflammation. But these vestiges of inflammatory action were confined to the duplicatures of the peritonæum enveloping these portions of the liver; and were probably occasioned by the increasing distention of the contained tubercles. These were the appearances on the outside.

A division was next made through the diseased portions of the liver, for the purpose of examining their internal structure; but nothing more could be observed than an irregular white mass, for the most part soft, and resembling the caseous contents of a scrophulous tumour. There was a disagreeable odour emitted from the incisions made into the tubercles, somewhat resembling what is perceived on puncturing the intestines; and here and there was observed an oozing of purulent matter, particularly on pressure being applied. The only vestiges of organization, or of life, were the blood-vessels, branches of the *vena portarum* entering into, and of the *vena cava hepatica* going out of the liver; the rest was one confused mass, white and soft throughout, except in several places tough ligamentous substances, the remains probably of obliterated arteries and veins. The right lobe exhibited few marks of disease externally; these consisting of several tubercles, of various colours, some white, some yellow, and some of a crimson tint; all distinct, and situated towards the left side. The internal appearance and structure were perfectly natural, except the different portions occupied by the tubercles; and the gall-bladder, full of healthy bile, was free from disease. Both the omenta were sound, as well as all the other abdominal contents, which have not been mentioned above. Within the abdomen were contained about three pints of yellowish serum.

I brought home, for the purpose of making a preparation, nearly one-fourth part of the liver, being that portion of it forming the tumour in the epigastrium, and appearing at one time to constitute the principal part of the disease; and this weighed two pounds and a half: consequently the whole must have amounted to ten or twelve pounds.

### II. *Dissection of the Hip.*

To ascertain the nature of the painful and distressing disease in this part, an incision was made through the great glutæal muscle, down to the capsule of the joint. This was found remarkably tense, being pressed upon in every direction by a cheesy kind of substance, adhering firmly to it, and contained within the joint; and, on the outside, exhibiting the appearance of a large tumour, of a very firm texture. This mass was partly hard and partly soft; its internal structure being chiefly formed of coagulable lymph and purulent matter, variously interspersed. In proportion as it approached towards the capsule, it was more firm, having there the appearance of perfect organization. Several large fragments, and innumerable spiculæ of bone, resembling exfoliations, were observable through every part. There was no head, neck, nor trochanter, to be found; these being all absorbed, and the upper extremity of the thigh-bone, to which they had originally been attached, was rough, and separated from the periosteum for the space of one inch downwards. The thigh, as before mentioned, was some inches shorter than the other. The dimension of the enlarged capsule, with this mass of disease adhering to it, was about six inches by four. The acetabulum ischii was free from disease.

### Remarks upon the above Case.

1. Dissection shewing that the aorta, and all its immediate branches, were free from disease, we were directly convinced that our opinion was erroneous, respecting the existence of aneurism. The strange and forcible pulsation felt in the epigastrium, can, however, readily be accounted for. By the systole of the heart, we know that a quantity of blood is uniformly propelled from the left ventricle into the aorta; but this vessel being already distended with arterial blood, a dilatation takes place, which is proportionate to the additional quantity thrown in, and synchronous with the action producing it. The artery feels the distension, and by its inherent elasticity, the contained fluid is driven through its ramifications over the whole body; to return by the venous system to its original source. Hence, nearly, the full force of the heart was continued even into the *abdominal aorta*, raising it against the superincumbent *liver;* and through the substance of this, imparting a regular pulsation from the portion of it, which lay directly over the artery to that forming the tumour mour in the epigastric region, and pressing against the integuments of this part. Hence the simultaneous stroke of the heart, and of the supposed aneurismal tumour. Possibly also the hepatic artery, or the short horizontal trunk of the cæliac artery itself, or both, may have contributed, as was suggested by Mr. Sandford, to form this deceitful pulsating motion.

Whenever cases of this nature have occurred, they appear always to have occasioned a contrariety of opinions; so that one may very justly say with Morgagni (De Causis & Sedibus Morbor. Epis. 39, Art. 20.) that it is not equally easy to avoid being deceived sometimes, when a body of some extent, which strikes against the hand, may either be a dilated artery, or a tumour lying upon an artery, which is not dilated. In proof of this observation, the same excellent writer, who was also a very experienced surgeon and profound anatomist, relates a case of what eventually turned out to be a suppurated jugular gland, being supposed by himself to be aneurism. (Loco citato.) Peter Tabarranus mentions, how greatly he was deceived by a pulsating tumour in the epigastric region, of the size of a fist, which he imagined to be a true aneurism; but which proved, on opening the body, to be nothing more than a schirrous tumour in the mesentery. (Obs. Anatom. ed. 2, n. 9.) So also the pulsation in the neck of Severinus's patient having puzzled many surgeons, and led them to pronounce it to arise from aneurism, was subsequently discovered, as he himself had predicted, to be occasioned by the action of the carotid artery under a bronchocele. (De Recond. Abs. Nat. lib. iv. cap. 6.) Hence Morgagni says, this circumstance, which every body sees so plainly that nobody can deny it (the pulsation of an artery), happening sometimes even in the external parts, holds surgeons in suspense, whether the disorder be aneurism or not. (Loc. cit.)

The same author relates a very singular species of pulsation, which proceeded from a spasmodic affection of some part of the alimentary canal, compressing the neighbouring blood-vessels. In the epigastrium was perceived a large hard body, striking and vibrating against the hand with great force; and resembling a large aneurismal tumour, which every now and then doubled its pulsations. Many surgeons had told the patient that the disorder was aneurism; but Morgagni observes, that it was much more easy to say what it was not than what it was. On examination, he discovered that the pulsating motion in the tumour, mour, compared with the action of the heart, did not correspond: and hence he was enabled with confidence to say, that there was no aneurism. (Epist. 39, Art. 18.) An abscess in the mediastinum, though a rare disease, has been found to exist; and has also been mistaken for an aneurism, from its having formed a tumour with pulsation, in or about the midddle of the sternum. (Lond. Med. Jour. Vol. II. p. 405.)

In the case I have related it was remarked, that before the man was confined to his bed, the heart's motion was felt sometimes in the epigastrium, and sometimes against the left side, according to the posture he happened to be in. This, I imagine, was owing to the liver, a weight of ten pounds, when he was erect, operating by its gravity upon the diaphragm, which was thus probably drawn down; and as the pericardium is naturally attached to that muscle, the heart would, of course, descend also. Hence the apex of this viscus would strike against the diaphragm; but, in a decumbent position, it would rise, which it probably did; and thus we perceived its action (the impulse being feeble), vibrating somewhere, near to the seventh true rib, but not striking against it.

The pain in the tumour, from hasty walking, during the time of its formation, was induced probably by the increased action of the vessels, in the part consequent on muscular exertion.

On considering the diseased state of the liver, it might appear singular that jaundice was not present. It happened, however, that the gall-bladder, and the ducts, subservient to this part and to the liver, were all along in a natural state; and as the bile, secreted in the right lobe, found a ready passage to these ducts, and hence into the duodenum, no absorption of it into the blood-vessels did, or was likely to take place. The organization of the other lobes was so far destroyed, that it is probable no bile had been secreted in them for a considerable time; no vestiges of that fluid being obvious on dissection, and the *poli biliarii* being annihilated and confounded with the general mass.

Diseases of the liver, much resembling the one I have described, are probably to be found related in other medical works, besides those I have mentioned; but the collection I have recourse to, though pretty considerable, only enables me to refer the inquisitive reader to the following:—Ed. Sandifort's Exercitat. Anatom. lib. ii. cap. 8. M. Baillie's Morbid Anatomy, 8vo. ch. ix.

2. To

2. To any one acquainted with the nature of caries of the hip-joint, it need hardly be observed, that the considerable retraction of the thigh in the present case, was owing to the action of the great muscles arising from the pelvis, and inserted into the *os femoris*, drawing up this latter part: a circumstance which invariably occurs, when the head of this bone, or the acetabulum, is destroyed.

The appearances on dissection, left no doubt as to the cause of the violent and unremitting pain in the hip, experienced by the patient. For the natural and healthy actions of this part being destroyed, and the capsule being amazingly distended, to allow of the formation and growth of the disease within; it must happen, of course, that great pain and suffering would be the consequence. Although the joints, in a healthy state, are capable of bearing the most violent motions without pain, yet, in a state of inflammation, they are well known to be most acutely sensible.

The remaining separated portions of the *os femoris*, as yet unobserved, may have constituted the spiculæ and fragments of bone diffused through the tumour; or they may have been formed by the arteries of the periosteum, or of the capsule, during the period of inflammation; as happens in other parts, when the natural action of the blood-vessels is altered or diseased. Are not the portions of bony matter found in *fungus hæmatodes*, and imparting the peculiar crackling feel, both on the outside, and when the diseased part is laid open, formed by a similar process?

